# Development of New Canned Chub Mackerel Products Incorporating Edible Seaweeds—Influence on the Minerals and Trace Elements Composition

**DOI:** 10.3390/molecules25051133

**Published:** 2020-03-03

**Authors:** Elsa F. Vieira, Cristina Soares, Susana Machado, M. Teresa Oliva-Teles, Manuela Correia, Maria João Ramalhosa, Ana Carvalho, Valentina F. Domingues, Filipa Antunes, Simone Morais, Cristina Delerue-Matos

**Affiliations:** 1 REQUIMTE—LAQV, Instituto Superior de Engenharia do Porto, Instituto Politécnico do Porto, R. Dr. António Bernardino de Almeida 431, 4249-015 Porto, Portugal; elsavieiraf@gmail.com (E.F.V.); cristina.md.soares@gmail.com (C.S.); su_tche@hotmail.com (S.M.); mtt@isep.ipp.pt (M.T.O.-T.); mmb@isep.ipp.pt (M.C.); mjr@isep.ipp.pt (M.J.R.); ana.p.santos.carvalho@gmail.com (A.C.); vfd@isep.ipp.pt (V.F.D.); cmm@isep.ipp.pt (C.D.-M.); 2 WEDOTECH—Companhia de Ideias e de Tecnologias, Lda, Rua do Seixal, 108, 4000-521 Porto, Portugal; filipasgantunes@gmail.com

**Keywords:** canned chub mackerel, seaweeds, minerals, Portuguese north-central coast

## Abstract

This study aimed to develop new canned chub mackerel products incorporating edible seaweeds (*Ascophyllum nodosum*, *Fucus spiralis*, *Saccorhiza polyschides*, *Chondrus crispus*, *Porphyra* sp. and *Ulva* sp.) harvested in the Portuguese North-Central coast, with simultaneous sensory improvement and minerals enrichment. Two processes were compared, namely the addition of seaweeds in i) the canning step and ii) in the brining step (as the replacement for salt). The concentrations of four macrominerals (Na, K, Ca and Mg), chloride, and twelve trace elements (Co, Cu, Fe, I, Li, Mn, Mo, Rb, Se, Sr, V and Zn) were determined by high-resolution continuum source flame atomic absorption spectrometry (HR-CS-FAAS) and inductively coupled plasma mass spectrometry (ICP-MS), respectively. Results showed that canned chub mackerel incorporating *C. crispus* and *F. spiralis* was found to be the preferred sensory option, also exhibiting contents enriched with Cl, Co, Cu, Fe, I, Li, Mg, Mn, Mo, Na, Rb, Se, and Sr. This effect was more pronounced when both seaweed species were added to replace the salt added in the brining step.

## 1. Introduction

Seaweeds are rich sources of minerals (8–40%) due to their capacity to absorb inorganic substances from the surrounding marine media and store them in their tissues [1]. Seaweed mineral compositions vary according to phylum, environmental (geographic location, season, wave exposure, seawater temperature, salinity, mineral levels in seawater, and the pH of seawater among other factors) and physiological conditions [2,3]. Moreover, a determining factor for mineral uptake by seaweeds is the content and type of polysaccharides in the cell wall structure [4]. In general, seaweeds present high concentrations of macrominerals (Ca, Mg, Na, P and K) and trace elements (Br, Co, Cu, Fe, I, Mn, Mo, Se and Zn), which significantly vary among red, brown and green species [4,5]. The accumulation of Mg and Fe seems to be prevalent in green seaweed species while red and brown seaweeds tend to accumulate higher concentrations of Na, K, Zn, Mn and I [4]. The health benefits of minerals from seaweeds have been reported in the literature [4,6]. For instance, seaweeds are reported as one of the most important vegetable sources of Ca, and their intake could be useful for reducing the risk of deficiency, particularly in pregnant women, teenagers, and elderly people [1]. Brown seaweed species are also known as good natural sources of I [4]; this trace element is important for the prevention of iodine deficiency disorders—the most prevalent cause of brain damage [7]; maintenance of thyroid function [4] and antioxidant and anticancer activities [8]. Matanjun et al. [9] reported [Na^+^]/[K^+^] ratios to be relatively low in seaweeds, e.g., 0.14–0.16, while values higher than 1 have been reported by other authors [6,10]. The World Health Organization (WHO) recommends a [Na^+^]/[K^+^] ratio close to one; the consumption of food products with a higher K content protects against high blood pressure and other cardiovascular risks [11].

According to a review by Santini et al. [12], there is not an officially accepted definition of a nutraceutical. The European Nutraceutical Association [13] defines nutraceuticals as nutritional products that provide health and medical benefits, including the prevention and treatment of disease. The presence of various bioactive compounds in seaweeds makes them highly applicable as nutraceuticals in the food and supplement industries. Depending on the seaweed species and the amount used in the formulation, the incorporation of seaweeds (or seaweed extracts) into food products can improve the shelf-life, nutritional, textural, organoleptic, sensory and health properties of the final products. Also, the synergistic effects of seaweeds with different ingredients could affect the health benefits of food products [14].

The effects of edible seaweeds and seaweed extracts addition to food properties were recently reviewed [4,8]. Seaweeds have found wide applicability in the meat industry and the effects on the mineral composition of meat products have been reported by several researchers. For instance, López-López et al. [15] evaluated the effects of three different types of edible seaweeds, Sea Spaghetti (*Himanthalia elongata*), Wakame (*Undaria pinnatifida*), and Nori (*Porphyra umbilicalis*), added at a concentration of 5.6% dry weight (dw) to the composition and antioxidant capacity of gel/emulsion meat systems. Results showed that the addition of seaweeds to meat products decreased the Na contents and increased the concentrations of K, Ca, Mg and Mn; the presence of Nori also caused an increase in the levels of Fe. In addition, López-López et al. [16] found that adding 5.5% dw of Sea Spaghetti produced low-sodium Frankfurters with better [Na^+^]/[K^+^] ratios and Ca contents. More recently, López-López et al. [17] found that adding 3% dw of Wakame seaweed produced beef patties with enhanced Na, K, Ca and Mg contents and a [Na^+^]/[K^+^] ratio close to 1. Nevertheless, scarce attention has been paid to the use of edible seaweeds as ingredients in canned fish products. In fact, to our best knowledge, a first attempt was made to add different seaweeds extracts (cochayuyo, sea lettuce, ulte, and red luche) as covering liquids to improve the lipid and sensory quality of canned Atlantic salmon [18]. Also, Hanjabam et al. [19] evaluated the impact of adding 3 or 5% of *Sargassum wightii* on the physicochemical, microbiological, antioxidant, and sensory qualities of tuna jerky.

The Atlantic Ocean is rich in high-value fish species with the Northeast Atlantic and Eastern Central Atlantic being included in the top seven marine fishing areas in the world [20]. The Atlantic chub mackerel (*Scomber japonicus*) is in the top 15 species caught by the European Union in 2015 and was the main species caught on the Portuguese coast in 2018, accounting for 24.9% of total fish caught [20]. According to the National Association of Manufacturers of Canned Fish (ANICP), 16,000 tons of canned chub mackerel were produced in Portugal in 2010 and about 60% of this production was focused on external markets. In addition to its high commercial interest, traditional canned chub mackerel products are well accepted by the consumers. Furthermore, canned products have a very long shelf-life, travel well and help in the management of food waste. Therefore, the development of new canned products incorporating seaweeds are of interest due to their considerable economic importance, broad acceptance by consumers, ease of transport and export, whilst constituting a nutritional strategy targeting the Recommended Dietary Allowance (RDAs) or Adequate Intake (AIs) of essential minerals, in particular iodine, which is a public health problem worldwide [7]. Therefore, in order to fill the gap concerning this issue, the present research was conducted (i) to develop new and sensorially acceptable canned chub mackerel products incorporating edible seaweeds harvested in the Portuguese North-Central coast; (ii) to evaluate how the addition of edible seaweeds can influence the mineral composition of the final products and (iii) to assess if such new products are an interesting dietary source of minerals. The concentration of four macrominerals (Na, K, Ca and Mg), Cl and twelve trace elements (Co, Cu, Fe, I, Li, Mn, Mo, Rb, Se, Sr, V and Zn) were thereby determined in both seaweeds and final canned formulation in this investigation.

## 2. Results and Discussion

### 2.1. Optimization of the Canned Chub Mackerel Products Incorporating Seaweeds

**Figure 1:**
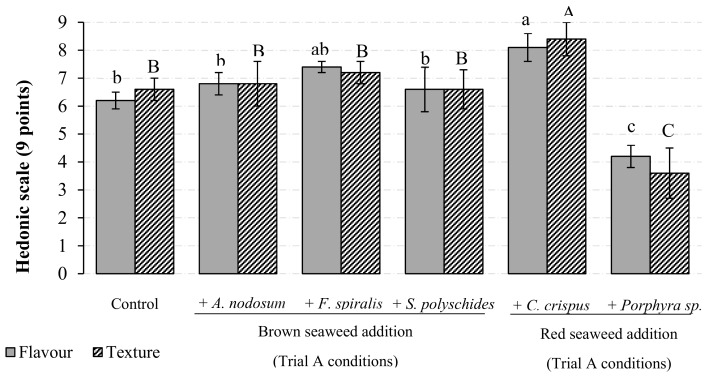
Sensory evaluation of flavour and texture (mean ± SD) of the canned mackerel produced by the traditional process (Control) and under the Trial A conditions with the incorporation of brown (*A. nodosum*, *F. spiralis*, *S. polyschides*) and red (*C. crispus*, *Porphyra* sp.) seaweed species. Attributes were evaluated by fifteen individuals using a hedonic scale (nine points), which ranged from ‘‘one—dislike extremely’’ to ‘‘nine—like extremely”. Bars labelled with different subscript (a–c) and superscript (A–C) letters have, respectively, mean values of flavour and texture significantly different at *p* < 0.05 (Tukey’s post hoc test).

The concentration of heavy metals (As, Cd, Pb and Sn) found in the five species harvested in the Portuguese North-Central coast were significantly (*p* < 0.05, 95% confidence) lower than the maximal levels authorized in seaweeds [21]. Thus, all species were used as ingredients in the formulation of canned chub mackerel products and included in the sensory analysis. Overall, the incorporation of seaweeds in canned chub mackerel had good acceptability in terms of general presentation, colour and odour parameters when compared to the Control. The only exception was the green species *Ulva* sp., which promoted a negative green colour in the fish fillet and impacted odour, hence it was excluded due to sensory evaluation of the flavour and texture parameters (Figure 1).

Results showed that for both attributes there were no significant differences (*p* > 0.05) in sensory scores of the Control and canned chub mackerel containing *A. nodosum, S. polyschides* and *F. spiralis*. Moreover, the incorporation of *Porphyra* sp. in canned chub mackerel was not sensorially preferred as indicated by the significant (*p* < 0.05) lower scores for flavour and texture in comparison to the Control. The canned chub mackerel incorporating *C. crispus* presented the best sensory results, according to sensory panel, this product has the “ability to mitigate the taste and soften the texture of chub mackerel”. Based on these results, *C. crispus* (red seaweed) and *F. spiralis* (brown seaweed) were the selected species for the development of new canned chub mackerel products. Both species are largely found in the Portuguese North-Central coast [22], which is positive in terms of sustainability. Further, the sensory analysis revealed that the addition of dehydrated seaweed was the preferable preparation process of seaweeds in comparison to frozen seaweed; the rehydration ratio (RR) of *C. crispus* and *F. spiralis* were respectively, 89% and 71%. In addition, based on the iodine content of *C. crispus* (146.6 ± 8.2 µg/g dw) and *F. spiralis* (272.7 ± 11.4 µg/g dw), it was concluded that 2 g of dry seaweeds (corresponding to approximately 20 g fresh weight (fw) of seaweeds) was enough to have a positive impact on the sensory attributes of the new canned chub mackerel products without exceeding the RDA of this element.

### 2.2. Macromineral Composition of the Canned Chub Mackerel Products Incorporating Seaweeds

**Figure 2:**
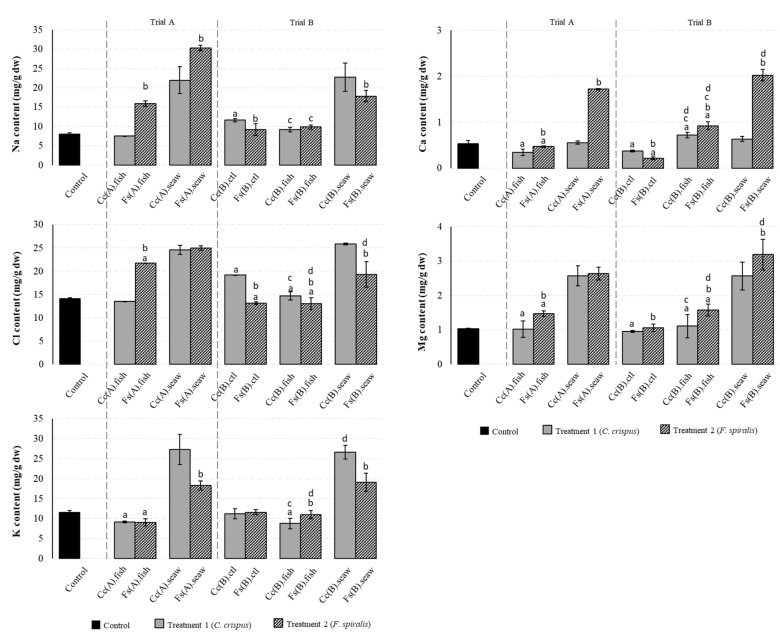
Macroelements composition (mg/g dw) of canned chub mackerel produced under the traditional process (Control) and incorporating *C. crispus* (Treatment 1—Trial A and B) or *F. spiralis* (Treatment 2—Trial A and B) seaweed species. Data represent the mean ± SD of six canned samples, analysed in duplicate. Statistical significance was determined by the Tukey’s post hoc test at: ^a^ *p* < 0.05 versus the Control sample; ^b^ *p* < 0.05 versus respective sample (fish/seaweed) obtained in Treatment 1 (in Trial A and B); ^c^ *p* < 0.05 versus Cc(B).ctl or Fs(B).ctl obtained in Treatment 1 and 2 (in Trial B), and ^d^ *p* < 0.05 versus respective fish sample (Cc(A).fish, Fs(A).fish) or seaweed sample (Cc(A).seaw or Fs(A).seaw) obtained in Treatment 1 and 2 (in Trial A).

**Figure 3:**
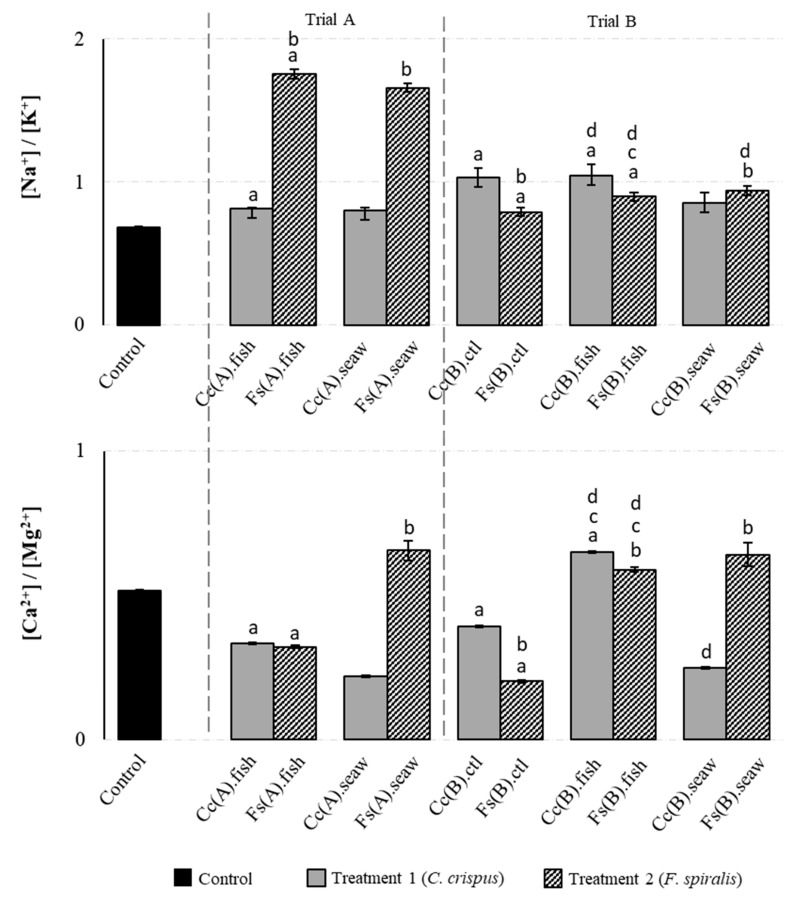
[Na^+^]/[K^+^] and [Ca^2+^]/[Mg^2+^] ratios of canned mackerel produced under the traditional process (Control) and incorporating *C. crispus* (Treatment 1—Trial A and B) or *F. spiralis* (Treatment 2—Trial A and B). Data represent the mean ± SD of six canned samples, analysed in duplicate. Statistical significance was determined by the Tukey’s post hoc test at: ^a^ *p* < 0.05 versus the Control sample; ^b^ *p* < 0.05 versus the respective sample (fish/seaweed) obtained in Treatment 1 (in Trial A and B); ^c^ *p* < 0.05 versus Cc(B).ctl or Fs(B).ctl obtained in Treatment 1 and 2 (in Trial B), and ^d^ *p* < 0.05 versus the respective fish sample (Cc(A).fish, Fs(A).fish) or seaweed sample (Cc(A).seaw or Fs(A).seaw) obtained in Treatment 1 and 2 (in Trial A).

The macromineral composition of canned chub mackerel products incorporating *C. crispus* (Treatment 1) and *F. spiralis* (Treatment 2), prepared under Trial A (addition of seaweeds in the canning step) and Trial B (addition of seaweeds as a replacement for salt added in the brining step and subsequent addition of seaweeds in the canning step) conditions, is presented in Figure 2; data from the traditional process (Control) are also included. Except for Na, the Control sample presented K, Ca and Mg contents within the range of those reported by the Portuguese food database [23]. When compared to the Control, the addition of *F. spiralis* in the canning step (Fs(A).fish) increased the Na, Cl and Mg contents by 99.5%, 53.4% and 42.9%, respectively, whereas the K and Ca concentrations were respectively decreased by 21.8% and 11.2%. In the same way, the addition of *C. crispus* (Cc(A).fish) decreased the Ca (35.9%) and K (20.9%) contents, although no significant effect was observed for the elements Na, Cl and Mg (*p* < 0.05). According to literature, seaweeds usually present an equilibrated [Na+]/[K+] ratio and although their Na levels are high in general, their consumption could contribute to an increase in K intake [4]. In this work, higher [Na^+^]/[K^+^] ratios were found in canned chub mackerel incorporating seaweeds in comparison with the Control (Figure 3). The ratios 0.82 (Cc(A).seaw) and 0.86 (Cc(B).seaw) were found for *C. crispus* treatment, whereas the ratios 0.94 (Fs(B).seaw) and 1.75 (Fs(A).seaw) were observed for *F. spiralis* treatment. These results are in agreement with the [Na^+^]/[K^+^] ratio of 1.46 reported for *F. spiralis* [6] but are significantly (*p* < 0.05) lower than the [Na^+^]/[K^+^] ratio of 1.34 reported for *C. crispus* [10]. Likewise, canned chub mackerel incorporating both seaweed species prepared under Trials A and B conditions, presented [Na^+^]/[K^+^] ratios ranging from 0.79 (Fs(B).ctl) to 1.75 (Fs(A).fish), which were significantly (*p* < 0.05) higher than those found in the Control, [Na^+^]/[K^+^] ratio of 0.69 (Figure 3). On the other hand, the ingestion of Ca and Mg are also correlated with cardiovascular health; Mg acts as a calcium antagonist on smooth muscle tone, thus causing vasorelaxation and blood pressure decrease [4]. The [Ca^2+^]/[Mg^2+^] ratio should remain close to 2.0 to prevent metabolic syndrome dysfunction in various tissues [24]. Based on the results presented in Figure 3, Trial B was the best treatment for improving the [Ca^2+^]/[Mg^2+^] ratio of canned chub mackerel incorporating seaweeds, corresponding to an increment of 14.0% (Fs(B).fish) to 26.2% (Cc(B).fish) in comparison to the Control.

### 2.3. Trace Elements Composition of the Canned Chub Mackerel Products Incorporating Seaweeds

The validation procedures using the fish muscle reference material (ERM^®^-BB422CRM) showed that ΔC ≤ UΔ, meaning that the measured mean values were not significantly different (*p* > 0.05) from the certified values (Table 1).

Thus, the inductively coupled plasma mass spectrometry (ICP-MS) methodology was reliably employed to quantify the mineral composition of developed canned chub mackerel products. The levels of trace elements in the developed canned chub mackerel products incorporating seaweeds are displayed in Table 2. With rare exceptions, the characterized products (Treatment 1 and Treatment 2—Trials A and B) exhibited higher (*p* < 0.05) concentrations of several elements in comparison to the Control; the improvements were as follows: Mn, 4223% for Fs(A).fish > Co, 1085% for Fs(A).fish > I, 994% for Fs(B).fish; Sr, 947% for Fs(A).fish > Mo, 279% for Cc(B).fish > Rb, 205% for Cc(B).fish > Li, 198% for Fs(A).fish > Cu, 129% for Cc(B).ctl > Fe, 47% for Cc(B).fish > Se, 30% for Fs(A).ctl. Notably, as chub mackerel is not a good source of Mn, the formulation of canned chub mackerel incorporating seaweeds might represent a good strategy to improve the general intake of this microelement. Hanjabam et al. [19] observed a 3.28-fold increase in Mn content when *Sargassum wightii* was incorporated in proportions of 5% in Tuna jerky batter formulation. Specifically, for the I contents variation, results suggest that the addition of *F. spiralis* as a replacement for salt added in the brining process and subsequent addition of seaweeds in the canning step (Fs(B).fish) can be the preferable treatment to enhance this mineral content in canned chub mackerel products. In this experiment, the I content of the final product (15.8 µg/g dw) was 10 times higher than those found in the Control sample (1.59 µg/g dw). Also, the formulated products presented higher levels of Rb, an essential ultra-trace element that biologically acts similar to K [25]. In contrast, the trace element V, with a role as material for bones and teeth and in the management of diabetes [26], was found in lower (*p* < 0.05) concentrations in canned chub mackerel products in which seaweeds were added in replacement of salt in the brining step (Cc(B).fish and Fs(B).fish). In fact, under this processing condition (Trial B), the concentration of V decreased by 72% and 76%, respectively in comparison to the Control. In the same way, the trace element Zn, a component of many enzymes, including those involved in neurotransmitter synthesis, energy metabolism, and collagen/elastin cross-linking [4], was markedly (*p* < 0.05) decreased in Cc(A).fish, Cc(B).fish and Fs(B).fish samples. Overall, the results suggested that migration effects of some elements from seaweeds to chub mackerel, and vice-versa, occurred without an obvious pattern. Thus, to better explain these effects, the mineral composition of the boiling water used in the replacement of salt added at the brining step (Trial B) should be also analysed. The mineral composition of the olive oil used in the filling step was analysed and the minerals were not detected.

### 2.4. Contribution of Canned Samples to Mineral RDA/AI

The assessment of mineral contributions to RDA or AI is essential for understanding the possible benefits and risks associated with the consumption of the developed canned chub mackerel products. Table 3 shows the contribution of EDI to RDA/AI derived from the consumption of canned chub mackerel incorporating *C. crispus* (Cc(B).fish) or *F. spiralis* (Fs(B).fish) formulated according to Trial B conditions. This treatment was selected due to the general high levels of macro and trace elements observed, as previously discussed. Results were expressed as %, considering the consumption of a can (which has approximately 60 g fw of mackerel and 20 g fw of seaweeds) by an adult with body weight (bw) of 70 kg. As shown in Table 3, the incorporation of *C. crispus* or *F. spiralis* increased (*p* < 0.05) the Na contribution to AI; the consumption of the whole canned product (fish plus seaweed) represented, respectively, 15.3% and 15.5% of the AI. This is not a positive result since Na intake generally exceeds the nutritional guidelines in industrialized countries and is associated with hypertension problems [27]. Notably, both seaweed species were regarded as a great source of I in the newly formulated products, accounting for the maximum value of 283% of RDA in the whole canned Fs(B).fish. product. Based on the iodine content of *C. crispus* (146.6 ± 8.2 µg/g dw) and *F. spiralis* (272.7 µg/g ± 11.4 dw), results suggest that the migration effects of I from seaweeds to chub mackerel were markedly higher (*p* < 0.05) in *F. spiralis* treatment. As a result, in comparison to the Control, the I contribution to RDA from consumption of isolated chub mackerel increased 10-fold for *F. spiralis* instead of 2-fold for the *C. crispus* treatment. These results suggest that the consumption of canned mackerel products incorporating *C. crispus* and *F. spiralis* exceed the RDA (150 µg/d) of this element, indicating that the quantity of seaweed used for its preparation should be reduced to ca. one third. On the other hand, when chub mackerel was incorporated with *C. crispus*, the Cu, Fe, Mn, Mo and Se contribution to RDA/AI were significantly (*p* < 0.05) increased; the whole canned product accounted the mean values of 13.9%, 12.6%, 7.0%, 22.7% and 95.4%, respectively. The increase in Cu and Fe RDAs is beneficial since both minerals are essential for the activity of several enzymes involved in energy metabolism and thermoregulation [5]. The increase of Se RDA contribution on the newly formulated products is positive since this essential trace element strongly influences inflammation and immune responses, with a relevant role in the protection of the oxidative state of lipid intermediates [5]. Also, the increase in Mo contribution to RDA, which was around 4 times in Cc(B).fish products is a positive result since this element forms part of the active sites of metalloenzymes which are involved in nitrogen assimilation, sulfur metabolism, and stress reactions [28]. In contrast, the contribution of Mg to RDA was significantly increased with the incorporation of *F. spiralis* (9.0%), the same was observed for Fe (9.5% RDA) and Mn (16.4% AI). Since chub mackerel is not a good source of Mn, the incorporation of *F. spiralis* presents a good opportunity to improve the general intake of this trace element, which is reported to have biological roles in antioxidant defence, energy production, immune response and regulation of neuronal activities [29].

## 3. Materials and Methods

### 3.1. Reagents and Analytical Solutions

The ultrapure water (18.2 MΩ cm resistivity) used to prepare all the aqueous solutions was from a Simplicity 185 water purification system (Millipore, Molsheim, France). Standard solutions of Na, K, Ca and Mg were prepared from 1000 mg/L stock solutions (Panreac Química SA, Barcelona, Spain) and were acidified with 0.5% (*v*/*v*) of 65% HNO_3_ (p.a., Sigma-Aldrich, Steinheim, Germany). Cesium chloride (0.1% *w*/*v*; Sigma-Aldrich, Darmstadt, Germany) was used as ionization buffer in Mg, K and Na analysis and lanthanum nitrate (0.1% *w*/*v*; Panreac Química SA, Barcelona, Spain) was added to the standards and samples solutions for Ca determinations. Potassium nitrate 99.0% and ammonium hydroxide solution (ACS reagent, 28–30% NH_3_ basis) were acquired from Sigma-Aldrich and silver nitrate 99.8% from Fluka, (Seelze, Germany). Calibration standards for inductively coupled plasma mass spectrometry (ICP-MS) analysis were prepared from a 10 mg/L multi-element standard solution (PlasmaCAL SCP-33-MS, SCP Science, Baie-D’Urfé, Quebec, H9X 4B6, Canada) and the internal standard solution was prepared by the appropriate dilution of the AccuTraceTM (AccuStandard^®^, New Haven, CT, USA) ICP-MS-200.8-IS-1 solution (100 mg/L of Sc, Y, In, Tb and Bi). Iodine (^127^I) and tellurium (^125^Te) isotopes were from Sigma-Aldrich. For analytical quality control purposes, the certified fish muscle matrix reference material ERM^®^-BB422 was used. All glassware and plastic material was soaked in 10% HNO_3_ for 24 h, rinsed with ultra-pure water and dried before use.

### 3.2. Preparation of Canned Chub Mackerel Incorporating Seaweeds

#### 3.2.1. Selection and Preparation of Seaweeds

Five species of seaweeds recognised as edible by Edible Seaweed-French, European Regulation and commonly found on the Portuguese coast [22] were analysed. Sampling included three species of brown seaweeds (*Ascophyllum nodosum* (*A. nodosum*), *Fucus spiralis* (*F. spiralis*) and *Saccorhiza polyschides* (*S. polyschides*)), two species of red seaweeds (*Chondrus crispus* (*C. crispus*) and *Porphyra* sp.), and one green species (*Ulva* sp). Seaweeds were collected in Aguda (Vila Nova de Gaia, Portugal) in October 2016 and prepared following the procedures described by Pereira [21] and Vieira et al. [31]. A preliminary study was carried out to evaluate the impact of the incorporation of seaweeds in the sensory performance of canned chub mackerel. For this purpose, two seaweeds pre-treatments were compared; the addition of frozen seaweed versus the addition of dehydrated (52 °C, for 6–8 h, using an Excalibur 9 Tray dehydrator, model 4926 T, USA) seaweed after a step of rehydration by immersion in tap water over a period of 10 to 15 min at room temperature. The rehydration ratio (RR) was calculated as described by Lewicki [32]. Secondly, an experiment was conducted to decide the best quantity of seaweeds to be incorporated in the canned samples; three important aspects were considered: (i) the visual impact of the seaweed in the final product; (ii) the sensory acceptance by potential consumers; and (iii) the contribution of seaweeds quantity to the recommended dietary dose of iodine (150 μg/day [30]). A group of 15 individuals from both genders tested the new canned chub mackerel incorporating seaweeds and the Control sample. The participants were from an internal panel selected from the research laboratories and the companies involved in this study, not sensory trained but regular consumers of canned chub mackerel (at least, one time a week). The questionnaire applied contained a hedonic scale (nine points), ranging from “one—dislike extremely” to “nine—like extremely” [33], to evaluate the sensory attributes flavour and texture; and a space at the end for general comments. Samples were marked with a numerical code attributed randomly, and the order of their tasting was decided randomly. Each sample was offered at room temperature and anonymously to all participants. Each participant was asked to judge a sample at a time and had access to water and unsalted crackers to help cleanse the palate before tasting the subsequent sample. The tasting method was standardized as follows: firstly, each participant was asked to open the can (with the same capacity of 60 g) and evaluate the colour uniformity and the aspect; secondly, each participant was asked to smell the product; finally, each participant used a fork to put the sample in a dish and assess the taste. Prior to sensory evaluation, the group participated in a cycle of lectures on the sensory attributes of in-oil mackerel. All participants were volunteers and signed a consent agreement. There were no vulnerable individuals involved in the tests and no personal data collection was necessary for this study.

#### 3.2.2. Canned Products Conception and Optimization

**Figure 4:**
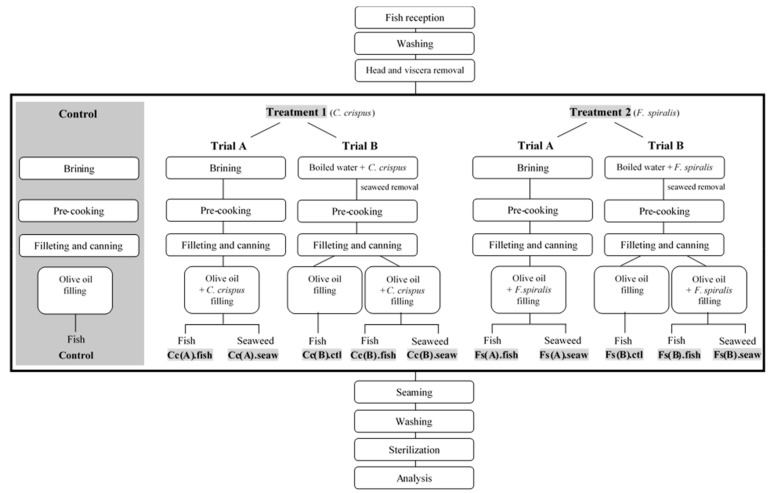
Scheme production for canned chub mackerel produced under the traditional process (Control) and for canned chub mackerel incorporating *C. crispus* (Treatment 1—Trials A and B) or *F. spiralis* (Treatment 2—Trials A and B) developed in this work. The respective fish (Cc(A).fish; Fs(A).fish; Cc(B).ctl; Cc(B).fish; Fs(B).ctl and Fs(B).fish) and seaweed (Cc(A).seaw; Fs(A).seaw; Cc(B).seaw and Fs(B).seaw) samples that resulted from Treatments 1 and 2 were analysed separately. For Trial B, the control samples Cc(B).ctl and Fs(B).ctl correspond to chub mackerel boiled with seaweed but without its addition in the filling step.

**Figure 5:**
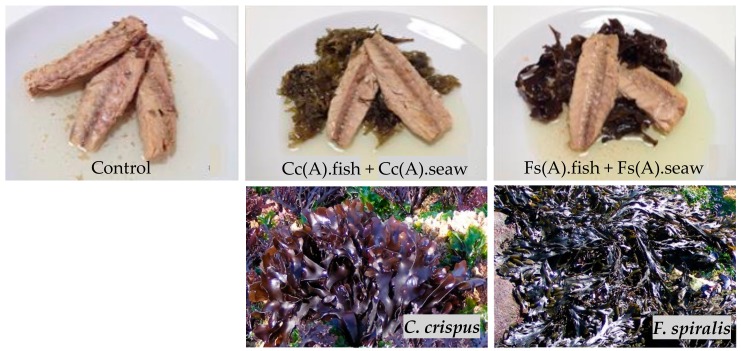
Samples of canned chub mackerel produced under the traditional process (Control) and canned chub mackerel incorporating *C. crispus* prepared under the Treatment 1, Trial A conditions (Cc(A).fish + Cc(A).seaw) or incorporating *F. spiralis* prepared under the Treatment 2, Trial A conditions (Fs(A).fish + Fs(A).seaw) (see Figure 4 for identification of samples). The bottom images of Figure 5 represent the *C. crispus* and *F. spiralis* seaweed species used for the preparation of the canned products.

The production of the canned chub mackerel incorporating seaweeds was carried on a traditional fish canning industry of the North of Portugal (Matosinhos, Portugal). The traditional process flowchart of the canned chub mackerel, designed as Control, is illustrated in Figure 4. The two crucial steps of the entire process are the brining step (usually made under 5° Bé), which is used to improve the organoleptic quality (i.e., flavour and texture) of fish, and the canning step, where olive oil is routinely used to fill the can. One of the objectives of the present work was to estimate the nutritional and sensory impact (and consumers` acceptance) of the addition of seaweeds to canned chub mackerel in the canning step (Trial A). The other goal of this work was to investigate the partial replacement of salt in the brining step by the addition of seaweeds (Trial B). For both purposes and based on the results of the preliminary sensory assessment, *C. crispus* and *F. spiralis* were the selected seaweed species. In Trial A, dehydrated *C. crispus* (Treatment 1) and dehydrated *F. spiralis* (Treatment 2) were added to chub mackerel in the canning step in the ratio of 2 g dw of seaweed/60 g fw (fresh weight) of fish. The respective fish (Cc(A).fish and Fs(A).fish for *C. crispus* and *F. spiralis*, respectively) and seaweed (Cc(A).seaw and Fs(A).seaw) samples that resulted from each treatment were analysed separately (Figure 5). In Trial B, dehydrated *C. crispus* (Treatment 1) and dehydrated *F. spiralis* (Treatment 2) seaweeds were boiled with chub mackerel in industrial recipients with 10 L of capacity for 20 min; the ratio of dehydrated seaweed/fish was 2 g dw of seaweed/60 g fw of fish. After this process, seaweeds were removed and then another portion of dehydrated seaweeds was added in the canning step, in the same proportion as previously mentioned. Samples coded as Cc(B).ctl and Fs(B).ctl correspond to chub mackerel boiled with dehydrated *C. crispus* or *F. spiralis*, respectively, but without its addition in the filling step. Fish and seaweed samples resulting from each treatment were analysed separately. Samples from Treatment 1 and Trial B were respectively codified as “Cc(B).ctl”, “Cc(B).fish” and “Cc(B).seaw”, whereas samples from Treatment 2 and Trial B were respectively codified as “Fs(B).ctl”, “Fs(B).fish” and “Fs(B).seaw”. Both Treatments 1 and 2 were compared with the Control. A total of 6 canned samples were developed for each Trial and for Control and further analyses were all performed, at least, in duplicate.

### 3.3. Chemical Characterization of Canned Samples

#### 3.3.1. Moisture and Ash Contents

Moisture and ash contents were determined by the 930.15 and 923.03 AOAC methods [34], respectively, and using 1.0 g of each fresh sample. Ash determination was performed placing the crucible with the dried samples in a muffle (Nabertherm B-180, Lilienthal, Germany) at 550 °C overnight and weighing after reaching room temperature [34]. All analyses were performed in triplicate.

#### 3.3.2. Microwave Digestion

Microwave-assisted digestion was performed in a Microwave Accelerated Reaction System (MARS-X, 1500, CEM, Matthews, NC, USA) configured with a 14-position carousel and equipped with pressure and temperature sensors. Approximately 0.20 g of dried sample was weighed to each of the microwave Teflon vessels and 10 mL of Suprapur HNO_3_ (65% *v*/*v*; Merck, Darmstadt, Germany) was added. Then, the microwave-assisted digestion proceeded for 35 min at 185 °C at 1200 W, as previously described by Torrinha et al. [35]. During operation, both temperature and pressure were monitored in a single vessel (control vessel). For iodine analysis, after microwave digestion and cooling to ca. 30 °C, a 2.5 mL aliquot of the acid digest was immediately added to 20 mL of 1:1 (*v*:*v*) ammonium hydroxide solution:water [36]. All samples were prepared, at least, in duplicate.

#### 3.3.3. Elemental Composition

The elements Na, K, Ca and Mg were analysed using a HR-CS-FAAS Analytik Jena ContrAA 700 (Analytik Jena, Jena, Germany) system equipped with a xenon short-arc lamp of 300 W (XBO 301, GLE, Berlin, Germany) and operating in a hot-spot mode using the conditions described by Oliveira et al. [37]. An air/acetylene oxidizing flame (Linde, Vila Nova da Telha, Portugal) was used and the equipment was coupled to an AS52S autosampler (Analytik Jena, Jena, Germany). The quantification of Cl was carried out according to the operating conditions of Machado et al. (2016). Briefly, a potentiometric titration (pH-meter Metrohm 780, Metrohm, Herisau, Switzerland) with a combined silver electrode (Ag-Titrode, Metrohm) and 0.1 mol/L AgNO3 standard solution was carried out with the aqueous extracts previously acidified to pH < 2 and the ionic strength adjusted with 0.1 mol/L KNO3. The elements Co, Cu, Fe, Li, Mn, Mo, Rb, Se, Sr, V and Zn were quantified by ICP-MS with an iCAP™ Q instrument (Thermo Fisher Scientific, Bremen, Germany) using the operational parameters described by Cabrita et al. (2016). I was also quantified by ICP-MS according to Costa Leite et al. [7]; the 127I isotope was monitored for analytical determination and the tellurium (125Te) isotope was used as an internal standard. The elements Ag, As, Be, Cd, Ce, Cr, Ni, Pb and Sn presented values inferior to the limit of detection for all samples (0.00033, 0.0017, 7.4 × 10^−5^, 0.0014, 8.1 × 10^−5^, 0.024, 0.055, 0.00023 and 0.00035 µg/g dw for Ag, As, Be, Cd, Ce, Cr, Ni, Pb and Sn, respectively) and were excluded from further discussion. All analyses were performed in triplicate.

#### 3.3.4. Quality Control

A fish muscle reference material, ERM^®^-BB422, was used for method validation and performance control. To assess the method performance, a minimum mass of 0.20 g of the certified material was digested and analysed at the same time as the samples for element measurement. The target parameters for certification were the mass fractions of As, Cd, Cu, Fe, Hg, I, Mn, Pb, Se, and Zn. Hg was not analysed in this work. The measured values for the chosen elements (As, Cd, Cu, Fe, I, Mn, Se, and Zn) were compared with the certified values following a procedure described by Linsinger [38] using Equation (1):

(1) $$\Delta_{C}\;\leq \;U_{\Delta }$$

where $\Delta_{C}=\left|MV-CRM\right|$ and $U_{\Delta }=2\;\times \;\surd \left(U_{CRM}^{2}+U_{MV}^{2}\right)$.

CRM is the certified reference material; MV is the measured value; UCRM is the uncertainty of the CRM value, and UMV is the uncertainty of the measured value. If ΔC ≤ UΔ then there is no significant difference between the measurement result and the certified value at a confidence level of about 95% in accordance with ISO/IEC Guide 98-3:2008 [39]. Major elements (Ca, K, Mg and Na) were also measured in the CRM by AAS. Cl analysis was previously validated for seaweed analysis according to Machado et al. [40] and Plácido et al. [41].

### 3.4. Assessment of Daily Intake

The estimated daily intake (EDI) of minerals from the consumption of canned chub mackerel products incorporating seaweeds was evaluated using Equation (2):

(2) $$\mathrm{EDI}\;\left(\mathrm{mg}/\mathrm{day}\right)=\mathrm{CM}\;\times \;\mathrm{MI}$$

where CM is the mineral concentration (mg/g fw) in fish or seaweed, and MI is the mass of the product ingested per day by a 70 kg body weight (bw) adult, i.e., 60 g fw of chub mackerel and 20 g fw of seaweeds corresponding to the mean weights of both ingredients in the packaged product. Considering that the olive oil in the packaged product is often discarded by consumers, its contribution for the EDI calculation was not accounted. The EDI values were used to calculate the percentage contribution of the canned product to minerals RDAs or AIs [30].

### 3.5. Statistical Analysis

All results were calculated on a dw basis and expressed as mean ± standard deviation (SD). The statistical analyses were performed using the data analysis program SPSS Inc., version 22.0 (SPSS Inc., Chicago, IL, USA). The Kolmogorov–Smirnov test was applied to verify whether the distribution of the variables was normal (*p* < 0.05). One-way analysis of variance (ANOVA) was carried out for comparing multiple samples, followed by Tukey’s post hoc test, and differences with *p* < 0.05 were considered significant. The observed statistical differences were coded as (^a^) the Control sample versus fish samples obtained in Treatments 1 and 2 (for both Trials A and B); (**^b^**) fish and seaweed samples obtained in Treatment 1 versus fish and seaweed samples obtained in Treatment 2 (for both Trials A and B); (^c^) Cc(B).fish and Fs(B).fish versus respective Cc(B).ctl and Fs(B).ctl obtained in Treatments 1 and 2 (in Trial B); and (^d^) fish and seaweed samples obtained in Trial A versus fish and seaweed samples obtained in Trial B (for both Treatments 1 and 2).

## 4. Conclusions

Canned chub mackerel incorporating *C. crispus* and *F. spiralis* was found to be sensorially acceptable by a small group of consumers and have enhanced content of certain elements, namely Cl, Co, Cu, Fe, I, Li, Mg, Mn, Mo, Na, Rb, Se, and Sr, which are often lacking or below recommended levels in regular diets. This effect was more pronounced when seaweeds were added as a salt replacement in the brining step. However, although the incorporation of seaweeds helped to balance the dietary [Na^+^]/[K^+^] and [Ca^2+^]/[Mg^2+^] ratios of canned products, they cannot be used for reducing the content of NaCl in canned chub mackerel. Furthermore, more extensive sensory tests of the formulated products are needed in order to increase the representativeness of the sensory data.

## Figures and Tables

**Table 1:** Comparison between the measured and the certified values for the Certified Reference Material ERM^®^-BB422.

Element	CRM (mg/kg dW)	U_CRM_ (mg/kd dw)	MV (mg/kg dw)	U_MV_ (mg/kg dw)	Δ_C_ (mg/kg dw)	U_Δ_ (mg/kg dw)
As	12.7	0.7	13.6	0.2	0.861	1.49
Cd	0.0075	0.0018	0.0068	0.00072	0.000720	0.00388
Cu	1.67	0.16	1.88	0.055	0.212	0.339
Fe	9.4	1.4	10.2	0.8	0.783	3.26
I	1.4	0.4	1.6	0.2	0.250	0.87
Mn	0.368	0.028	0.356	0.028	0.0118	0.0792
Se	1.33	0.13	1.18	0.13	0.151	0.365
Zn	16.0	1.1	14.0	0.2	2.018	2.23

CRM—certified reference material; MV—measured value; U_CRM_—uncertainty of the certified value; U_MV_—uncertainty of the measured value. $\Delta_{C}\;=\left|MV-CRM\right|$; $U_{\Delta }=2\times \sqrt{\left(U_{CRM}^{2}+U_{MV}^{2}\right)}$.

**Table 2:** Trace elements composition (µg/g dw) of canned mackerel produced under the traditional process (Control sample); canned mackerel incorporating *C. crispus* (Treatment 1) or *F. spiralis* (Treatment 2) seaweeds produced under the Trial A and B conditions.

	Co	Cu	Fe	I	Li	Mn	Mo	Rb	Se	Sr	V	Zn
**Control**	0.02 ± 0.01	3.04 ± 0.25	31.1 ± 3.57	1.59 ± 0.18	0.11 ± 0.02	0.39 ± 0.03	0.13 ± 0.02	1.51 ± 0.07	2.29 ± 0.26	3.80 ± 1.04	0.15 ± 0.01	19.6 ± 0.26
**Trial A**												
**Treatment 1**												
Cc(A).fish	0.09 ± 0.01 ^a^	3.63 ± 0.49 ^a^	41.4 ± 3.56 ^a^	5.26 ± 0.23 ^a^	0.26 ± 0.01 ^a^	4.10 ± 0.29 ^a^	0.36 ± 0.02 ^a^	4.55 ± 0.13 ^a^	2.68 ± 0.12 ^a^	13.2 ± 0.61 ^a^	0.39 ± 0.02 ^a^	11.4 ± 1.80 ^a^
Cc(A).seaw	0.03 ± 0.01	4.60 ± 0.10	37.0 ± 4.36	133 ± 13.3	0.13 ± 0.01	3.20 ± 0.24	0.24 ± 0.02	1.41 ± 0.03	1.82 ± 0.23	4.03 ± 0.36	0.10 ± 0.01	17.1 ± 0.73
**Treatment 2**												
Fs(A).fish	0.21 ± 0.01 ^ab^	2.78 ± 0.14 ^b^	34.5 ±9.39 ^ab^	9.92 ± 1.07 ^ab^	0.34 ± 0.02 ^ab^	16.7 ± 0.44 ^ab^	0.12 ± 0.01 ^b^	2.89 ± 0.11 ^ab^	2.71 ± 0.19 ^a^	36.0 ± 3.0 ^ab^	0.49 ± 0.02 ^ab^	30.6 ± 2.79 ^ab^
Fs(A).seaw	0.04 ± 0.01 ^a^	2.82 ± 0.19 ^b^	26.3 ± 2.16 ^b^	47.9 ± 2.70 ^b^	0.15 ± 0.02	3.42 ± 0.15	0.17 ± 0.02 ^b^	1.35 ± 0.06	2.88 ± 0.22 ^b^	17.4 ± 1.71 ^b^	0.15 ± 0.01 ^b^	14.0 ± 1.28 ^b^
**Trial B**												
**Treatment 1**												
Cc(B).ctl	0.04 ± 0.01	6.97 ± 0.60 ^a^	37.1 ± 3.38 ^a^	2.51 ± 0.16 ^a^	0.16 ± 0.01	0.42 ± 0.03	0.46 ± 0.04 ^a^	1.48 ± 0.08	2.98 ± 0.32	3.50 ± 0.22	0.04 ± 0.02 ^a^	20.1 ± 1.90 ^a^
Cc(B).fish	0.12 ± 0.01 ^ac^	5.69 ± 0.38 ^acd^	45.7 ± 2.95 ^acd^	3.18 ± 0.19 ^acd^	0.33 ± 0.02 ^acd^	5.79 ± 0.35 ^acd^	0.48 ± 0.03 ^ad^	4.57 ± 0.64 ^ac^	2.39 ± 0.23 ^a^	16.8 ± 1.21 ^ac^	0.27 ± 0.01 ^acd^	12.4 ± 0.33 ^acd^
Cc(B).seaw	0.04 ± 0.01	5.50 ± 0.17 ^d^	48.3 ± 3.29 ^d^	102 ± 8.44 ^d^	0.13 ± 0.01	4.82 ± 0.41 ^d^	0.34 ± 0.07 ^d^	1.27 ± 0.06	2.35 ± 0.11 ^d^	9.45 ± 0.62 ^d^	0.07 ± 0.01 ^d^	21.1 ± 2.16 ^d^
**Treatment 2**												
Fs(B).ctl	0.06 ± 0.01	2.87 ± 0.11 ^ab^	31.1 ± 3.16 ^b^	1.60 ± 0.18 ^b^	0.17 ± 0.17 ^ab^	0.33 ± 0.02 ^ab^	0.10 ± 0.03 ^b^	1.43 ± 0.04	2.72 ± 0.22 ^a^	3.44 ± 0.88	0.04 ± 0.01 ^a^	17.9 ± 0.75 ^a^
Fs(B).fish	0.14 ± 0.04 ^acd^	2.78 ± 0.47 ^ab^	35.6 ± 2.40 ^ab^	15.8 ± 1.74 ^abcd^	0.20 ± 0.09 ^abcd^	14.3 ± 1.36 ^abcd^	0.10 ± 0.02 ^b^	1.63 ± 0.78 ^ad^	1.97 ± 0.11 ^abcd^	27.8 ± 2.18 ^abcd^	0.15 ± 0.08 ^bcd^	10.5 ± 0.93 ^abcd^
Fs(B).seaw	0.05 ± 0.02	3.01 ± 0.33 ^bd^	25.5 ± 1.83 ^b^	54.1 ± 4.31 ^bd^	0.17 ± 0.05 ^b^	4.03 ± 0.36 ^bd^	0.06 ± 0.01 ^bd^	1.31 ± 0.39	2.63 ± 0.23	20.2 ± 4.76 ^b^	0.05 ± 0.03 ^d^	16.6 ± 1.37 ^bd^

Trial A (addition of seaweeds in the canning step), Trial B (addition of seaweeds as a replacement for salt added in the brining step and subsequent addition of seaweeds in the canning step). Data represent the mean ± SD of six canned samples, analysed in duplicate. Statistical significance was determined by the Tukey’s post hoc test at: ^a^ *p* < 0.05 versus the Control sample; ^b^ *p* < 0.05 versus the respective sample (fish/seaweed) obtained in Treatment 1 (in Trial A and B); ^c^ *p* < 0.05 versus Cc(B).ctl or Fs(B).ctl obtained in Treatment 1 and 2 (in Trial B), and ^d^ *p* < 0.05 versus the respective fish sample (Cc(A).fish, Fs(A).fish) or seaweed sample (Cc(A).seaw or Fs(A).seaw) obtained in Treatment 1 and 2 (in Trial A).

**Table 3:** Recommended dietary allowance (RDA; mg/d) or adequate intake (AI; mg/d) and contribution (%) of the estimated daily mineral intake (EDI) to RDA or AI derived from the consumption of canned mackerel incorporating *C. crispus* (Cc(B).fish) or *F. spiralis* (Fs(B).fish) prepared under Trial B conditions.

		EDI (% of RDA or AI)						
		Treatment 1				Treatment 2		
Element	RDA or AI^•^	Control	Cc(B).fish	Cc(B).seaw	serving	Fs(B).fish	Fs(B)seaw	serving
Ca	1000	1.1	1.4	0.1	1.6 *	1.8	0.4	2.3 *
Cl	2300	12.3	12.8	2.3	15.1 *	11.4	1.7	13.1 *
Cu	0.9	6.8	12.6 *	1.2	13.9 *	6.2	0.7	6.8 *
Fe	8	7.8	11.4 *	1.2	12.6 *	8.9	0.6	9.5 *
I	0.15	21.2	42.4 *	135	178 *	211*	72.1	283 *
Li	1#	0.2	0.7	0.0	0.7 *	0.4	0.0	0.4 *
Mg	420	4.9	5.3	1.2	6.5 *	7.5*	1.5	9.0 *
Mn	2.3	0.4	6.4	0.5	7.0 *	15.9 *	0.4	16.4 *
Mo	0.045	5.8	21.2 *	1.5	22.7 *	4.5	0.3	4.8 *
Na	1500	10.6	12.3 *	3.0	15.3 *	13.1 *	2.4	15.5 *
K	4700	4.9	3.7	1.1	4.9 *	4.7	0.8	5.5 *
Se	0.055	83.3	86.9 *	8.5	95.4 *	71.5 *	9.6	81.1 *
Zn	11	4.9	3.1	0.5	3.6 *	2.6	0.4	3.0 *

A serving dose of 20 g dw of mackerel (corresponding to 60 g fw of fish with a mean moisture content of 63.1% ± 5.1%) and 2 g dw. of seaweed *C. crispus*/*F. spiralis* (corresponding to 20 g fw. of seaweed with a mean moisture content of 22.3% ± 2.4%) were considered for a 70 kg bw adult. EDI was evaluated using Equation (2) presented in Section 3.4. Assessment of daily intake. Recommended dietary allowances (RDA) and adequate intakes (AI^•^) for men with 31–50 years old [30]. # Provisional RDA for a 70 kg adult [30]. “Serving” is the sum of mackerel and seaweed, taking into consideration that a person will consume both ingredients in the packaged product. * Significance was determined at * *p* < 0.05, versus the Control sample.
